# Pharmacy Professionals' Dispensing Practice, Knowledge, and Attitude towards Emergency Contraceptives in Gondar Town, Northwestern Ethiopia: A Cross-Sectional Study

**DOI:** 10.1155/2017/8754126

**Published:** 2017-07-10

**Authors:** Sewunet Admasu Belachew, Dawit Kumilachew Yimenu, Begashaw Melaku Gebresillassie

**Affiliations:** ^1^Department of Clinical Pharmacy, School of Pharmacy, College of Medicine and Health Sciences, University of Gondar, Gondar, Ethiopia; ^2^Department of Social Pharmacy, School of Pharmacy, College of Medicine and Health Sciences, University of Gondar, Gondar, Ethiopia

## Abstract

**Background:**

Pharmacy professionals, as the most available members of medical team, have an important role in educating patients about the effective and appropriate use of contraceptives. The purpose of this study was to assess pharmacy professionals' dispensing practice, knowledge, and attitude towards emergency contraceptives use in Gondar town, northwestern Ethiopia.

**Methods:**

An institution based cross-sectional study was employed from May 14 to June 14, 2016, on 60 pharmacy professionals, who have been working in 8 randomly selected pharmacies and 6 drug stores. The collected data was entered to and analyzed using Statistical Packages for Social Sciences (SPSS) version 20.

**Result:**

More than half 33 (55.0%) of the participants were druggist with 5–9 years of experience. About 56 (93.3%) of the participants knew about the dosing schedule (when and how much to take) and side effects of emergency contraceptives. More than two-thirds of the participants (39, 65%) agreed that the existence of emergency contraceptives is a positive thing and considered their use is ethical (42, 63.3%). The majority of participants (51, 85%) also reported that they counsel all women when dispensing emergency contraceptive pills (ECPs).

**Conclusion:**

This study revealed that knowledge, attitude, and dispensing practice of emergency contraceptives are very good even though there were variations with respect to different factors. Findings suggested that additional training and proper counseling technique on emergency contraceptives will improve the service delivery.

## 1. Introduction

Emergency contraception pills (ECPs) are safe and effective oral products in minimizing the risk of pregnancy after unprotected sexual intercourse. There are kinds of hormonal contraception, containing high doses of estrogen and progestin (ethinyl estradiol plus levonorgestrel) or progestin only (levonorgestrel) which are 75%–89% effective in preventing pregnancies when taken within 72 hours (3 days) after sexual intercourse [1, 2]. Studies conducted on Yuzpe regimen of emergency contraception show that the 72-hour window may be restrictive and have extended effectiveness up to 5 days; however the earlier a woman gets the emergency contraception, the more effective the medication will be [3, 4].

The ECP can prevent pregnancies which were unwanted and attributable to contraceptive failure, lack or incorrect use of other contraceptive methods, or coerced sex. An effective use of ECP has also an advantage of reducing the rate of unsafe abortion [5].

According to the report of WHO, every year an estimated 80 million women have pregnancies which were unplanned and among these pregnancies many of them are aborted. Among these cases women from Sub-Saharan countries constitute the larger proportion [6]. The rate of unplanned pregnancies worldwide is still very high [7]. The unplanned pregnancies resulting in labor for some can result in induced abortion for others, which is still a major cause of maternal deaths [8].

Unintended/unwanted pregnancies (UP) are an important cause for many maternal deaths. Pregnancies that occur too early, too frequently, or too late can lead to illness at the time of pregnancy and complications during the time of birth [9]. Globally, there are about 287,000 maternal deaths in 2010, of this 99% (284,000) account for developing countries. At the country level, 10 countries comprised 60% of the global maternal deaths, out of this Ethiopia ranked 7th by 9,000 maternal deaths in 2010 [10]. In developing countries, about 40% of all pregnancies are unintended [9]. In Sub-Saharan Africa (SSA) 39% of pregnancies are unintended, which ranged from 30% in Western Africa to 59% in Southern Africa in 2008 [11]. The consequence of unwanted childbearing could affect both the born child and the mother. Women with UP have lower health outcomes such as increased risk of adverse birth outcomes, delayed recognition of pregnancy, gestational diabetes, and hypertension during pregnancy leading to hospitalization during pregnancy compared to women with intended pregnancies [12, 13].

A high proportion (15%–30%) of hospital gynecological admissions in many African regions result from complications of unsafe induced abortion [14]. Nowadays, access to ECPs is increasing and it will help to reduce unintended pregnancies and induced abortions through pharmacy professionals provision without the need for a physician's prescription [8]. Pharmacists can play a substantial role in promoting the use of ECPs. Pharmacies are ideal settings for dispensing ECPs for many reasons. First, women who need ECPs can purchase them from pharmacies since they are sold as an over-the-counter drug in the country. Women's access to ECPs over the counter makes use of ECPs more effective because they can get them as soon as they need them [15, 16]. Secondly, pharmacies are ideal settings for dispensing ECPs because of flexible hours of service and because they are open in nightfall and weekends when most health professionals are not available. Hence some pharmacies are known to have private spaces for counseling, and it makes the situation easier to provide confidential counseling for ECP clients and makes this role feasible [16].

In Ethiopia, although the practice of pharmacy is embracing a paradigm shift from product oriented service to patient oriented pharmaceutical service, pharmacists are not actively involving inpatient treatment decision; instead the pharmacist responds to prescribing errors long after the decision has been made and without having direct clinical knowledge of the patient, which often leads to unacceptable level of treatment outcomes. In line with this, cognizant of the global shift in pharmacy education and practice, schools of pharmacy in Ethiopia revised their curriculum in 2008 to be more patient focused. The curriculum aims to produce general pharmacists with the necessary knowledge, skills, and attitude to provide clinical pharmacy services [17]. Pharmacists need to have sufficient knowledge and be imbued with a positive attitude towards this type of contraceptives in order to fulfill their potential roles in encouraging use of ECPs [18]. Emergency contraceptive pills access as well as their use and availability is influenced by pharmacy personnel's knowledge and attitudes towards them [19]. Studies in some other settings have also revealed that women's access to emergency contraception may be obstructed by lack of knowledge and negative attitudes among pharmacy and health care staff [20–24]. To the best of our knowledge and search, no study was conducted in Ethiopia to explore pharmacy professionals dispensing practice, knowledge, and attitudes on ECP. The aim of the present study was to assess pharmacy professionals knowledge, attitude, and practice towards emergency contraceptives and to identify possible barriers to access these essential drugs.

## 2. Methods and Materials 

### 2.1. Study Area and Design

An institution based cross sectional study was employed to assess dispensing practice, knowledge, and attitude towards emergency contraceptives among pharmacy professionals in Gondar town. Gondar, which is located in the northwest part of the country 750 km away from the capital city Addis Ababa, is one of the ancient and historical towns in Ethiopia. The town has one referral and teaching hospital, one private general hospital, a number of health centers and private clinics, and 53 MROs (34 drug stores and 19 pharmacies).

The source population includes all pharmacy professionals who are working in medication retail outlets located in Gondar town, whereas the study includes all pharmacy professionals who are working at selected medication retail outlets.

### 2.2. Sample Size Determination and Sampling Procedure

Among the 19 pharmacies and 34 drug stores in the town, 8 drug stores and 5 pharmacies were selected using simple random sampling technique. These constituted about 25% of the respective MROs selected as a compromise between representativeness and feasibility. Then, all the available pharmacy professionals from selected medication retail outlets who fulfilled the inclusion criteria were included in this study.

### 2.3. Inclusion and Exclusion Criteria

All voluntary pharmacy professionals who are working at medication retail outlets were included in the study, whereas those who are working at store/stock management and administrative positions were excluded.

### 2.4. Study Variables

The independent variables include sex, religion, pharmacy training, years of practice, family planning training, and emergency contraceptives training. The dependent variables were pharmacy professionals dispensing practice, knowledge, and attitude towards emergency contraceptives.

### 2.5. Data Collection and Quality Assurance

The instrument used for data collection was adopted from previous similar studies and pretested on 5 pharmacy professionals who were not included in the final analysis and the necessary modifications were instituted before the actual data collection was started. The data were collected by four principal investigators through interviewer-administered questionnaires by explaining the questions for those who were unable to understand. The investigators who participated in the data collection were given all the necessary training about the instrument and appropriate ways of approaching the pharmacy professionals and gaining their permission for filling the questionnaire prior to the data collection process.

### 2.6. Operational Definition

#### 2.6.1. Emergency Contraception Pills (ECPs)

Hormonal contraceptives contain high doses of estrogen and progestin (ethinyl estradiol plus levonorgestrel) or progestin only (levonorgestrel) which are 75%–89% effective in preventing pregnancies when taken within 72 hours (3 days) after sexual intercourse.

### 2.7. Data Processing and Analysis

The collected data using quantitative method were cleaned and entered to and analyzed using IBM SPSS Statistics for Windows, version 20.0. Sociodemographic characteristics, knowledge, attitude, and dispensing practice towards the pharmacy professionals were described using frequencies, percentage, mean, and cross-tabulations.

### 2.8. Ethical Consideration

This study was conducted after ethical clearance was gained from research and ethics review committee of School of Pharmacy and the Clinical Directorate of Gondar University Referral Hospital. All of the study participants were provided with clear explanations about the purpose of the study and asked for their consent to participate in the study. They were also informed that participation was voluntary and if they want they could withdraw from the study at any stage. The data collected was kept confidential and, in addition, patient identifiers were not used and the collected data was used for the purpose of the study only.

## 3. Results

### 3.1. Sociodemographic Characteristics of Respondents

Out of the total interview guides/questionnaires of 68 sample pharmacy professionals who were interviewed in this study 60 were included in the analysis. Eight respondents were excluded from the analysis due to incompleteness making the response rate 88.2%. About two-thirds (60%) of the respondents in this study were males and in the age range of 20–30 years (65%). More than half (55%) of the respondents were druggist in their educational qualification with 5–9 years of experience. More than half the respondents (51.7%) had experience of training about family planning and emergency contraceptives (61.7%) (Table 1).

### 3.2. Knowledge about Emergency Contraceptive Pills

Almost all (93.3%) of the respondents knew about the dosing schedule (when and how much to take) of emergency contraceptives (Table 2). There was no much difference between male and female pharmacists with regard to EC knowledge. A cross-tabulation was performed to determine the association of knowledge with professional training experience and years of practice. The results showed that all of the 19 (100%) of the pharmacists knew about the dosing schedule while 31 out of the 33 (93.9%) of the druggists and 6 out of the 8 (75%) of the pharmacy technicians knew about the dosing schedule (Table 2). A positive relationship was found between knowledge and years of practice; all of the study participants with >10 years of practice knew about ECs dosing schedule while 96.9% and 87.5% of the participants with 5 to 10 and <5 years of practice knew about their dosing schedule, respectively (Table 3).

About 86.7% of the respondents knew about the mechanism of action of emergency contraceptives. The result from cross-tabulation showed that this has also a positive relation with professional training experience of the study participants; hence almost all (94.7%) pharmacists knew about the mechanism of action of ECs while 87.9% and 62.5% of druggists and pharmacy technicians knew about their mechanism of action, respectively. A cross-tabulation finding also revealed that there is a positive relationship between pharmacy professionals knowledge and years of practice they have (Table 3).

### 3.3. Attitudes Regarding Emergency Contraception

The majority of the study participants (65%) agreed that the existence of emergency contraceptives is a positive thing and they believe its use is ethical (63.3%). Regarding information and availability, more than half of the respondents (58.3%) agreed that all sexually active women should be aware of emergency contraceptives while 75% of the respondents agreed that routine information about ECPs should be included in contraceptive counseling. More than two-thirds (68.3%) of the study participants agreed about the availability of ECs OTC, while about 18.3% of the respondents disagreed. Regarding regulatory restrictions, only 33.3% of the respondents agreed that only women should have access to ECs (Table 4).

More than two-thirds (73.3%) of the respondents agreed that EC should be available for nonprescription use with appropriate counseling by pharmacy professionals and they feel competent instructing patients about the appropriate use of ECs (68.4%). Regarding the risk behavior associated with use of ECs, the majority of the respondents (53.3%) agreed that increased knowledge of emergency contraceptives will result in more unsafe sex. Similarly they agreed that nonprescription EC use will also result in more unsafe sex (61.7%). However, about 58.3% of the respondents agreed nonprescription EC use will discourage regular contraception use (Table 4).

### 3.4. Dispensing Practices of Emergency Contraceptives

The overall dispensing practice of emergency contraceptives at community pharmacies was very good. About 85% of the respondents reported that they counsel all women when dispensing ECPs to them. Among those respondents more than two-thirds (70%) reported that they give counseling about the possible drug interactions and contraindications to emergency hormonal contraceptives (78.3%) when dispensing them. Along with this more than two-thirds (70%) of respondents counsel about possible side effects of EHCs and how/when to take ECs if the woman is breast feeding (68.3%) (Table 5).

More than half of (68.3%) of the respondents reported that they counsel about the effect of ECs on sexually transmitted infections (STIs) and give special counseling for women with special needs like hearing/visual problems and mental health problem (58.3%) (Table 5).

### 3.5. Association of Dispensing Practice with Years of Experience

The overall cross-tabulation result showed dispensing practice of emergency contraceptives has a positive relation with years of practice (experience). All (100%) of the pharmacy professionals with more than 10 years of experience counsel about possible drug interactions, contraindications, and possible side effects. Moreover, all of them also counsel about the effect of ECs on sexually transmitted diseases and give special counseling for women with special needs (Table 6).

## 4. Discussion

For countries like Ethiopia, which have a very high rate of unintended pregnancies and abortion rates, the contribution of community pharmacists to the provision of emergency contraceptives and their appropriate use is very magnificent in reducing the incident [25].

This study was conducted to identify knowledge, attitudes, and dispensing practices of community pharmacy professionals in Gondar town regarding emergency contraceptive pills. To our knowledge, this study is the first study which provides preliminary data about community pharmacists' knowledge towards EC, not only in Gondar but also in Ethiopia. This study showed that general knowledge about hormonal emergency contraception among the community pharmacy professionals was found to be high (93.3%). The result of the present study was in line with a similar study conducted in Turkish where the report showed awareness rate of 91% and with that of study conducted in Nicaragua where all of the pharmacy professionals reported being aware of emergency contraceptive pills [8, 26]. However, the results of the present study were higher than a study conducted in south Dakota and Nigeria where the levels of knowledge of the respondents were low and average, respectively [18, 27]. Moreover, their scores were not different with regard to sex, whereas there were differences with regard to their professional training and years of practice. This might be attributed to the fact that pharmacy professional who has trained in bachelor of pharmacy (Bpharm) (pharmacists) program has a greater opportunity to take many courses about ECs and had many practical attachments in community pharmacies during their professional training compared to the corresponding druggists and pharmacy technicians.

The difference observed with regard to years of practice may be attributed to the fact that pharmacy professionals who work for many years will have many encounters compared to the corresponding less experienced pharmacy professionals in dispensing ECs and in the meanwhile will update themselves based on the enquiries from the clients. Apart from this it might be attributed to the fact that those very experienced pharmacy professionals might have opportunities to participate in different workshops and training about family planning and EC use compared to the corresponding less experienced pharmacists.

Pharmacy professionals attitude and acceptance regarding the existence of ECs will have a huge implications for patients access and use of emergency contraceptives. Most of the respondents in this study have a good attitude towards ECs. The majority (65%) of the study participants agreed that the existence of emergency contraceptives is a positive thing and the use of emergency contraceptives is ethical (63.3%). Regarding information and availability, the majority (58.3%) of the respondents agreed that all sexually active women should be aware of emergency contraceptives, while 75% of the respondents agreed that routine information about ECPs should be included during contraceptive counseling. These findings were in agreement with a study conducted on Turkish pharmacy technicians [8]. About 46.6% of the respondents agreed to the question “for adult fertile women to keep ECP at home is positive”; however this was lower than the result from the Turkish pharmacy technicians in which 60% of the pharmacy technicians agreed that it is a positive thing for adult fertile women to keep ECP at home [8]. However, despite their positive attitude towards the existence of emergency contraceptives and provision of information, pharmacy staff had certain concerns on the regulatory restrictions and the risks associated with them. For example, in case of regulatory restrictions, thirty-eight of the respondents (63.4%) agreed that ECPs should only be available to those >18 years of age and 33.3% of the respondents agreed that only women should have access to ECs while 50% of the respondents agreed for ECs to be available to everyone and 16.7% were neutral. This result was in agreement with a research conducted in South Africa in which a significant number of pharmacists doubt about their appropriateness, either on demand or in advance, for women younger than 18, and admitted denying minors access to emergency contraceptives [24].

With regard to the risk behavior associated with the use of ECs, the majority of the respondents (53.3%) agreed that increased knowledge of emergency contraceptives will result in more unsafe sex and about 73.3% of the respondents agreed that EC should be available for over-the-counter use with required counseling by pharmacy professionals. These results were in line with the findings reported from the study conducted on Turkish pharmacy technicians in which 63% of the pharmacy technicians showed concerns about the risk behaviors related to the increased knowledge and availability of the ECP, such that increased knowledge of ECP would result in more unsafe sex [8] Similarly 61.7% of the respondents agreed that nonprescription EC use will result in more unsafe sex and about 58.3% of the respondents agreed that nonprescription EC will discourage regular contraception use. These results were also in agreement with the findings reported from a research conducted on Nicaraguan pharmacists in which the majority of study participants were highly concerned that the availability of these pills might encourage sexual risk-taking behaviors such as lack of condom use and increase the risk of transmission of HIV and other sexually transmitted diseases [26]. 68.4% of the total respondents agreed that they feel competent instructing patients about the appropriate use of ECs. This finding was almost in agreement with the finding from Turkish pharmacy technicians (60%) [8].

The majority (85%) of the respondents reported that they counsel all women when dispensing ECPs to them. These findings also showed that the counseling practice is varied among the respondents based on their educational qualification and years of practice. This might be explained by their knowledge, in which pharmacists and those respondents who practiced for many years could have better knowledge and experience compared to the corresponding druggists/pharmacy technicians and less experienced respondents.

As a limitation, it is difficult to generalize the finding of this study to other areas in Ethiopia, since pharmacy professionals level of health care services and medical knowledge in Gondar town is better compared to other areas in the country. Also, knowledge and certain attitudes among pharmacy professionals who failed to participate in the study may be different from those of the respondents.

## 5. Conclusion

Emergency contraceptives are very important contraceptive options nowadays in Ethiopia where the incidence of unwanted pregnancies and abortion rate is high. As ECP awareness increases the demand for the availability of ECPs and the use of ECP is likely to continue to increase in the country, pharmacy professionals are likely to continue to play significant roles in providing ECP to those who need it to prevent unwanted pregnancies because of their flexible hours of operation and because these medication retail outlets are closer to people when compared to other health care facilities. It is important for the pharmacy professionals to be aware of and knowledgeable about emergency contraception to help women use it effectively. They are also likely to be the point of contact with the health care system for not only providing ECs but also for information about it. The findings of this study also revealed that they could benefit from additional training in proper counseling and ECs use.

## Figures and Tables

**Table 1:** Distribution of respondents by sociodemographic characteristics, Gondar, 2016.

Variable		Frequency (%)
Age (in years)	20–30	39 (65)
	31–40	14 (23.3)
	41–50	4 (6.7)
	>50	3 (5)

Sex	Male	36 (60)
	Female	24 (40)

Religion	Orthodox	46 (76.7)
	Catholic	1 (1.7)
	Muslim	11 (18.3)
	Protestant	2 (3.3)

Educational qualification	Pharmacy technician	8 (13.3)
	Druggist	33 (55.0)
	Pharmacist	19 (31.7)

Years of practice (in years)	1–4	24 (40.0)
	5–9	33 (55.0)
	≥10	3 (5.0)

Trained in family planning	Yes	31 (51.7)
	No	29 (48.3)

Trained in emergency contraceptives	Yes	23 (38.3)
	No	37 (61.7)

**Table 2:** Percentage distribution of respondents knowledge and cross-tabulation of knowledge with professional training, Gondar, 2016.

Knowledge *n* (%)			Educational qualification *n* (%)		
			Pharmacist	Druggist	Pharmacy technician
Do you know the dosing schedule (when and how much to take) for ECs?	Yes	56 (93.3)	19 (31.6)	31 (51.6)	6 (10)
	No	4 (6.7)	0 (0)	2 (3.3)	2 (3.3)
Do you know the mechanism(s) of action of ECs?	Yes	52 (86.7)	18 (30)	29 (48.3)	5 (8.3)
	No	8 (13.3)	1 (1.6)	4 (6.6)	3 (5)
Do you know the side effects of ECs?	Yes	56 (93.3)	18 (30)	31 (51.6)	7 (11.6)
	No	4 (6.7)	1 (1.6)	2 (3.3)	1 (1.6)
Do you know contraindications to the use of ECs?	Yes	48 (80.0)	17 (28.3)	25 (41.6)	6 (10)
	No	12 (20.0)	2 (3.3)	8 (13.3)	2 (3.3)
Do you know how much effective ECs are in preventing pregnancy?	Yes	46 (76.7)	17 (28.3)	23 (38.3)	6 (10)
	No	14 (23.3)	2 (3.3)	10 (16.6)	2 (3.3)

**Table 3:** Cross-tabulation of respondents knowledge with their year of practice, Gondar, 2016.

Knowledge		Years of practice		
		<5 years (*n* = 24)	5–10 years (*n* = 33)	>10 years (*n* = 3)
Do you know the dosing schedule (when and how much to take) for ECs?	Yes	21 (87.5%)	32 (97%)	3 (100%)
	No	3 (12.5%)	1 (3%)	0 (0%)
Do you know the mechanism(s) of action of ECs?	Yes	20 (83.3%)	29 (87.9%)	3 (100%)
	No	4 (16.7%)	4 (12.1%)	0 (0%)
Do you know the side effects of ECs?	Yes	21 (87.5%)	32 (97%)	3 (100%)
	No	3 (12.5%)	1 (3%)	0 (0%)
Do you know contraindications to the use of ECs?	Yes	17 (70.8%)	28 (84.9%)	3 (100%)
	No	7 (29.2%)	5 (15.1%)	0 (0%)
Do you know how much effective ECs are in preventing pregnancy?	Yes	16 (66.7%)	27 (81.8%)	3 (100%)
	No	8 (33.3%)	6 (18.2%)	0 (0%)

**Table 4:** Percentage distribution of respondents attitude towards emergency contraception, Gondar, 2016.

variables	Strongly disagree *n* (%)	Disagree *n* (%)	Neither agree nor disagree *n* (%)	Agree *n* (%)	Strongly agree *n* (%)
Reproductive health					
Existence of emergency contraceptives is positive	5 (8.3)	9 (15)	7 (11.7)	31 (51.7)	8 (13.3)
Use of ECs is ethical	3 (5)	9 (15)	10 (16.7)	32 (53.3)	6 (10)
Teenagers and youngsters can take responsibility for the use of ECP	8 (13.3)	17 (28.3)	11 (18.3)	21 (35)	3 (5)
ECP gives women increased sexual safety	16 (26.7)	14 (23.3)	8 (13.3)	21 (35)	1 (1.7)
ECP increases women's control of reproduction	7 (11.7)	13 (21.7)	7 (11.7)	22 (36.7)	11 (18.3)
All sexually active women should be aware of ECP	9 (15)	9 (15)	7 (11.7)	24 (40)	11 (18.3)
ECP should be as well-known as Condoms	5 (8.3)	14 (23.3)	12 (20)	22 (36.7)	7 (11.7)
Routine information about ECP should be included in contraceptive counseling	5 (8.3)	5 (8.3)	5 (8.3)	29 (48.3)	16 (26.7)
Information and availability					
All sexually active men should be aware of ECP	7 (11.7)	7 (11.7)	7 (11.7)	29 (48.3)	10 (16.7)
Information of ECP should be included in sex education in school	3 (5)	11 (18.3)	4 (6.7)	26 (43.3)	16 (26.7)
To have ECP available OTC is Positive	5 (8.3)	6 (10)	8 (13.3)	30 (50)	11 (18.3)
For adult fertile women to keep ECP at home is positive	14 (23.3)	13 (21.7)	5 (8.3)	20 (33.3)	8 (13.3)
Regulatory restrictions					
ECP should be sold only to women	14 (23.3)	16 (26.7)	10 (16.7)	15 (25)	5 (8.3)
ECP should be sold only to those over 18 years of age	4 (6.7)	10 (16.7)	8 (13.3)	22 (36.7)	16 (26.7)
ECP should be sold only to women with specific characteristics such as rape victims	10 (16.7)	17 (28.3)	8 (13.3)	13 (21.7)	12 (20)
Risk behavior					
Men will be less willing to use condom when they know about ECP	14 (23.3)	14 (23.3)	7 (11.7)	18 (30)	7 (11.7)
Increased knowledge of ECP results in more unsafe sex	11 (18.3)	12 (20)	5 (8.3)	20 (33.3)	12 (20)
ECP can make it more difficult for women to refuse unprotected intercourse	12 (20)	14 (23.3)	8 (13.3)	17 (28.3)	9 (15)
EC should be available for nonprescription use WITH required counseling by pharmacist	4 (6.7)	6 (10)	6 (10)	30 (50)	14 (23.3)
Nonprescription EC will promote unsafe sex	4 (6.7)	10 (16.7)	9 (15.0)	27 (45)	10 (16.7)
Nonprescription EC will discourage regular contraception use	4 (6.7)	14 (23.3)	7 (11.7)	23 (38.3)	12 (20)
I am uncomfortable dispensing EC for moral or religious reasons	12 (20)	13 (21.7)	13 (21.7)	16 (26.7)	6 (10)
I feel competent instructing patients regarding the appropriate use of EC	3 (5)	5 (8.3)	11 (18.3)	34 (56.7)	7 (11.7)

**Table 5:** Percentage distribution of respondents dispensing practice of ECs, Gondar, 2016.

Variables		Frequency (%)
Do you counsel all women when dispensing ECPs to them?	Yes	51 (85.0)
	No	9 (15.0)
Do you counsel possible drug interactions with EHCs?	Yes	42 (70.0)
	No	18 (30.0)
Do you counsel contraindications to EHCs?	Yes	47 (78.3)
	No	13 (21.7)
Do you ask if the woman has any problems that might affect the absorption of EHC?	Yes	31 (51.7)
	No	29 (48.3)
Do you counsel what to do if vomiting occurred after taking the pill?	Yes	44 (73.3)
	No	16 (26.7)
Do you counsel how much and when to take ECs?	Yes	51 (85.0)
	No	9 (15.0)
Do you counsel possible side effects of EHCs and what to do if they occur?	Yes	42 (70.0)
	No	18 (30.0)
Do you counsel what to do if signs of ectopic pregnancy occur after taking ECPs? (if any severe lower abdominal pain occurs after taking this medicine)	Yes	39 (65.0)
	No	21 (35.0)
Do you counsel about the use of continued contraception?	Yes	44 (73.3)
	No	16 (26.7)
Do you counsel how/when to take ECs if the woman is breast feeding?	Yes	41 (68.3)
	No	19 (31.7)
Do you counsel the effect of ECs on next period?	Yes	39 (65.0)
	No	21 (35.0)
Do you counsel about the effect of ECs on sexually transmitted infections (STIs)?	Yes	41 (68.3)
	No	19 (31.7)
Do you give special counseling for women with special needs (hearing/visual problems, mental health problem) while dispensing ECs to them?	Yes	35 (58.3)
	No	25 (41.7)

**Table 6:** Cross-tabulation of respondents dispensing practice of ECs with their years of experience, Gondar, 2016.

Variables		Years of practice		
		<5 years (*n* = 24)	5–10 years (*n* = 33)	>10 years (*n* = 3)
Do you counsel all women when dispensing ECs?	Yes	20 (83.3%)	29 (87.9%)	2 (66.7%)
	No	4 (16.7%)	4 (12.1%)	1 (33.3%)
Do you counsel possible drug interactions with ECs?	Yes	16 (66.7%)	23 (69.7%)	3 (100%)
	No	8 (33.3%)	10 (30.3%)	0 (0%)
Do you counsel contraindications to ECs?	Yes	18 (75%)	26 (78.8%)	3 (100%)
	No	6 (25%)	7 (21.2%)	0 (0%)
Do you ask if the woman has any problems that may affect the absorption of ECs?	Yes	14 (58.3%)	15 (45.5%)	2 (66.7%)
	No	10 (41.7%)	18 (54.5%)	1 (33.3%)
Do you counsel what to do if vomiting occurs after taking the pills?	Yes	15 (62.5%)	26 (78.8%)	3 (100%)
	No	9 (37.5%)	7 (21.2%)	0 (0%)
Do you counsel how much and when to take ECs?	Yes	22 (91.7%)	28 (84.8%)	3 (100%)
	No	2 (8.3%)	5 (15.2%)	0 (0%)
Do you counsel possible side effects of ECs and what to do if they occur?	Yes	17 (70.8%)	22 (66.7%)	3 (100%)
	No	7 (29.2%)	11 (33.3%)	0 (0%)
Do you counsel what to do if signs of ectopic pregnancy occur after taking ECs?	Yes	17 (70.8%)	19 (57.6%)	3 (100%)
	No	7 (29.2%)	14 (42.4%)	0 (0%)
Do you counsel about the use of continued contraception?	Yes	16 (66.7%)	25 (75.8%)	3 (100%)
	No	8 (33.3%)	8 (24.2%)	0 (0%)
Do you counsel how and when to take ECs if the woman is breast feeding	Yes	16 (66.7%)	22 (66.7%)	3 (100%)
	No	8 (33.3%)	11 (33.3%)	0 (0%)
Do you counsel the effect of ECs on next period?	Yes	16 (66.7%)	20 (60.6%)	3 (100%)
	No	8 (33.3%)	13 (39.4%)	0 (0%)
Do you counsel the effect of ECs on sexually transmitted diseases?	Yes	13 (54.2%)	25 (75.8%)	3 (100%)
	No	11 (45.8%)	8 (24.2%)	0 (0%)
Do you give special counseling for women with special needs?	Yes	12 (50%)	20 (60.6%)	3 (100%)
	No	12 (50%)	13 (39.4%)	0 (0%)
